# Processing of Genome 5′ Termini as a Strategy of Negative-Strand RNA Viruses to Avoid RIG-I-Dependent Interferon Induction

**DOI:** 10.1371/journal.pone.0002032

**Published:** 2008-04-30

**Authors:** Matthias Habjan, Ida Andersson, Jonas Klingström, Michael Schümann, Arnold Martin, Petra Zimmermann, Valentina Wagner, Andreas Pichlmair, Urs Schneider, Elke Mühlberger, Ali Mirazimi, Friedemann Weber

**Affiliations:** 1 Department of Virology, University of Freiburg, Freiburg, Germany; 2 Department of Microbiology, Tumor and Cell Biology, Karolinska Institutet, Stockholm, Sweden; 3 Centre for Microbiological Preparedness, Swedish Institute for Infectious Disease Control, Solna, Sweden; 4 Department of Virology, University of Marburg, Marburg, Germany; 5 Immunobiology Laboratory, Cancer Research UK, London Research Institute, London, United Kingdom; Institut Pasteur Korea, Republic of Korea

## Abstract

Innate immunity is critically dependent on the rapid production of interferon in response to intruding viruses. The intracellular pathogen recognition receptors RIG-I and MDA5 are essential for interferon induction by viral RNAs containing 5′ triphosphates or double-stranded structures, respectively. Viruses with a negative-stranded RNA genome are an important group of pathogens causing emerging and re-emerging diseases. We investigated the ability of genomic RNAs from substantial representatives of this virus group to induce interferon via RIG-I or MDA5. RNAs isolated from particles of Ebola virus, Nipah virus, Lassa virus, and Rift Valley fever virus strongly activated the interferon-beta promoter. Knockdown experiments demonstrated that interferon induction depended on RIG-I, but not MDA5, and phosphatase treatment revealed a requirement for the RNA 5′ triphosphate group. In contrast, genomic RNAs of Hantaan virus, Crimean-Congo hemorrhagic fever virus and Borna disease virus did not trigger interferon induction. Sensitivity of these RNAs to a 5′ monophosphate-specific exonuclease indicates that the RIG-I-activating 5′ triphosphate group was removed post-transcriptionally by a viral function. Consequently, RIG-I is unable to bind the RNAs of Hantaan virus, Crimean-Congo hemorrhagic fever virus and Borna disease virus. These results establish RIG-I as a major intracellular recognition receptor for the genome of most negative-strand RNA viruses and define the cleavage of triphosphates at the RNA 5′ end as a strategy of viruses to evade the innate immune response.

## Introduction

The efficacy of the innate immune response against virus infections is highly dependent on a rapid production of interferons and other cytokines. To achieve this, all nucleated cells are endowed with a cytoplasmic signaling cascade leading from the detection of intruded virus particles to the expression of cytokines, including type-I interferons (IFN-α/β) [1], [2]. IFN-α/β directly induce an antiviral state that alleviates the virus burden but also initiate adaptive immune responses [3]. Viruses that block expression of IFN-α/β are therefore often highly virulent [4], [5].

An important group of pathogens causing emerging and re-emerging diseases are viruses with a negative-stranded RNA genome (NSVs). Prominent examples are influenza viruses (family *Orthomyxoviridae*), Ebola virus (*Filoviridae*), rabies virus (*Rhabdoviridae*), Nipah virus (*Paramyxoviridae*), Lassa virus (*Arenaviridae*), and several members of the *Bunyaviridae* family e.g. Rift Valley fever virus, Hantaan virus, or Crimean-Congo hemorrhagic fever virus (Table 1). These pathogens can cause rapid, systemic and often fatal illnesses which are characterized either by a fulminant pneumonia and multi-organ failure or by a severe hemorrhagic fever [6]–[9].

**Table 1:** Negative-strand RNA viruses (NSVs).

Family	Genome organisation	Representative members
*Filoviridae*	nonsegmented	Zaire Ebola virus (ZEBOV)
*Paramyxoviridae*	nonsegmented	Nipah virus (NiV), measles virus (MeV)
*Rhabdoviridae*	nonsegmented	rabies virus, vesicular stomatitis virus
*Bornaviridae*	nonsegmented	Borna disease virus (BDV)
*Arenaviridae*	2 segments	Lassa virus (LASV)
*Bunyaviridae*	3 segments	Rift Valley fever virus (RVFV; Phlebovirus)
		Hantaan virus (HTNV; Hantavirus)
		Crimean-Congo hemorrhagic fever virus (CCHFV; Nairovirus)
*Orthomyxoviridae*	6 to 8 segments	influenza A virus (FLUAV)

NSV particles consist of a lipid envelope and a genome which is encapsidated by the viral nucleocapsid protein and polymerase. The genome is present either as one continuous strand of RNA (nonsegmented NSVs), or is divided into two, three, or up to eight segments. Upon infection of a host cell, the NSV genome first needs to be transcribed into mRNAs in order to furnish the proteins necessary for virus replication. In the course of these events, the incoming nucleocapsids must be unwrapped and the genomic RNA exposed. Cells, in turn, are able to sense the infection and activate an innate immune response. They immediately synthesize cytokines, most prominently the antivirally active type I interferons, but also other pro-inflammatory cytokines and chemokines [10].

The main intracellular sensors to recognize viral RNA structures and trigger cytokine synthesis are the RNA helicases RIG-I and MDA5 [11]–[13]. Until very recently, it was assumed that the only viral agonist of these pathogen recognition receptors (PRRs) is double-stranded RNA (dsRNA) which is generated as a by-product of genome replication. However, we have recently found that NSVs are exceptional in not producing substantial amounts of dsRNA [14]. Instead, the genomic single-stranded RNA (ssRNA) bearing a 5′ triphosphate group was shown to be sufficient to activate RIG-I dependent IFN-α/β induction [15], [16]. The importance of RIG-I for recognizing genomic RNA has been demonstrated for influenza virus, rabies virus and vesicular stomatitis virus [15], [16], but for none of the other NSVs.

Here, we tested genomic RNA isolated from highly pathogenic NSVs to investigate whether activation of RIG-I is a common denominator. Our results not only establish RIG-I as the main intracellular sensor for the genome of both nonsegmented and segmented NSVs, but also show that some viruses are able to escape RIG-I-dependent cytokine induction by a hitherto unrecognized stealth strategy.

## Results

### Genomic RNAs from highly virulent nonsegmented NSVs activate the IFN system

**Figure 1:**
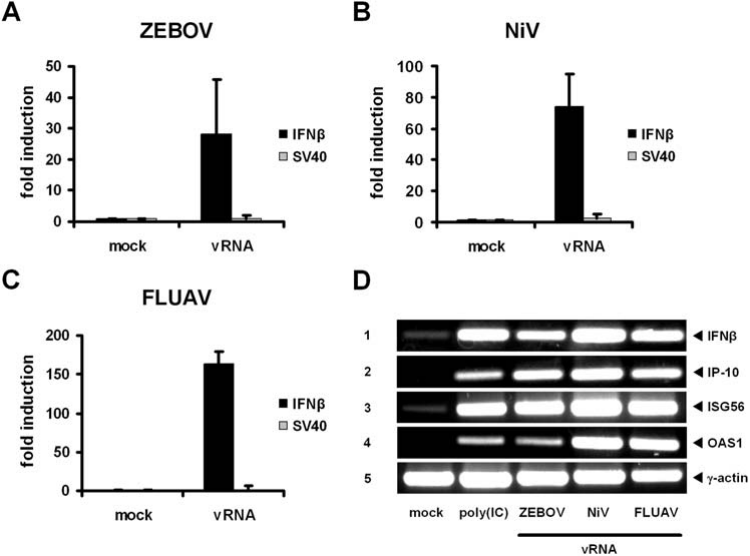
Genomic RNAs of ZEBOV and NiV activate the IFN response similar to FLUAV RNA. Human 293T cells were transfected with luciferase reporter plasmids to measure activation of the inducible IFN-β promoter and the constitutively active SV40 promoter, respectively. At 6 h post-transfection, cells were either mock treated or transfected with 1 µg viral genomic RNA (vRNA) of ZEBOV (A), NiV (B), or FLUAV (C). After overnight incubation, cells were lysed and promoter activities were normalised to the mock-induced samples. Mean values and standard deviations from 3 independent experiments are shown. (D) Detection of mRNAs for IFN-β (panel 1) and the IFN-stimulated genes IP-10, ISG56, and OAS (panels 2 to 4). Detection of γ-actin mRNA served as control (panel 5). 293T cells were transfected with 1 µg vRNA or 5 µg of the dsRNA analog poly(IC) and monitored 18 h later for gene upregulation by RT-PCR analysis.

We tested the genomic RNAs of Zaire Ebola virus (ZEBOV, family *Filoviridae*) and Nipah virus (NiV, family *Paramyxoviridae*) for the capability to activate an innate immune response. RNA isolated from influenza A virus (FLUAV) particles was used as positive control. As assessed in a reporter assay (Fig. 1A and B), transfection of human 293T cells with RNAs isolated from ZEBOV and NiV particles (vRNA) resulted in strong activation of the IFN-β promotor, in a manner similar to FLUAV vRNA (Fig. 1C). The constitutively active SV40 promoter, by contrast, was not activated by vRNAs, indicating specificity for the IFN system. Furthermore, RT-PCR analysis showed that treatment of cells with vRNAs resulted in a transcriptional upregulation of the IFN-β gene as well as the IFN-stimulated genes IP-10, ISG56 and OAS1 (Fig. 1D), thus confirming our results with the reporter system. We concluded from these data that genomic RNAs of ZEBOV and NiV are potent elicitors of an IFN response.

### IFN induction depends on RIG-I and the 5′ triphosphate group of genomic RNAs

**Figure 2:**
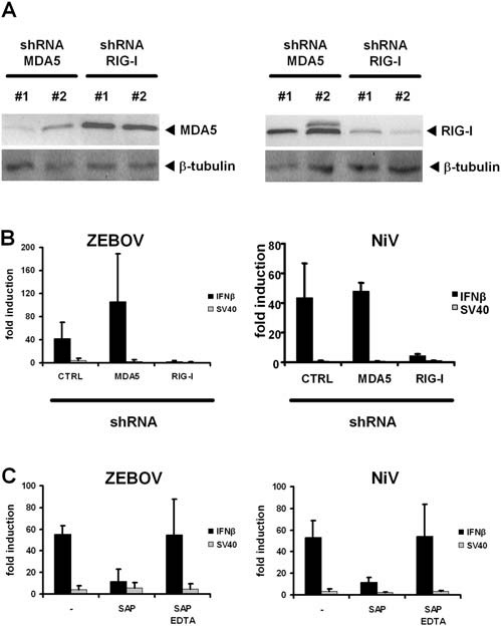
IFN induction by NSV vRNAs depends on RIG-I and the 5′ triphosphate group. (A) Verification of knockdowns. Human 293T cells were treated with retroviral shRNA constructs directed against either RIG-I or MDA5, and cotransfected with expression constructs for HA-tagged MDA5 (left panels) or GFP-fused RIG-I (right panels). Western blot analysis using antibodies against the respective fusion tags is shown. Detection of cellular β-tubulin was used as an internal control. (B) Effect of shRNA knockdowns on IFN induction by viral RNAs, using the reporter constructs and RNA transfection protocols as described for Fig. 1A. The negative control shRNA construct (CTRL) targets the heat shock 70 interacting protein and was tested to have no effect on IFN induction (data not shown). (C) Genomic RNAs from ZEBOV and NiV were either mock treated, treated with SAP, or treated with SAP in the presence of the phosphatase inhibitor EDTA. IFN-β reporter assays and RNA transfections were performed as described for Fig. 1A. Mean values and standard deviations from 3 independent experiments are shown.

MDA5 and RIG-I are the main intracellular PRRs driving an antiviral innate immune response against RNA viruses [13]. To investigate which of these factors is required for the recognition of ZEBOV and NiV RNA, we established RNA interference (RNAi) in 293T cells, using short hairpin RNAs (shRNAs) expressed by retroviruses. Initially, for each target two different retroviral shRNA constructs were tested for their efficiency to knock down gene expression. In Fig. 2A it is demonstrated that the shRNA constructs MDA5 #1 and RIG-I #2 significantly and specifically reduced target expression levels, whereas MDA5 #2 and RIG-I #1 had more modest effects. Therefore, in all subsequent experiments, we used MDA5 #1 and RIG-I #2 for gene-specific knockdowns. As a next step, we pretreated cells with these constructs, incubated them for 5 days to establish RNAi, seeded them again and transfected them with viral RNAs. The IFN-β promoter assay was used as a representative measure of cytokine induction. Fig. 2B shows that IFN induction by ZEBOV and NiV vRNAs was affected when RIG-I was downregulated. By contrast, MDA5 knockdown had no negative effect but sometimes increased IFN induction to some - statistically not significant - extent.

RIG-I-dependent responses to FLUAV vRNA were previously shown to critically depend on the presence of a 5′ triphosphate group [16]. In agreement with this, genomic RNAs from ZEBOV and NiV particles lost their IFN-inducing activities when treated with shrimp alkaline phosphatase (SAP). This effect could be inhibited by EDTA, excluding an unspecific effect of SAP on IFN induction (Fig. 2C).

These data suggest that the 5′triphosphate group is the decisive molecular pattern triggering a RIG-I-dependent IFN response to genomic RNAs of ZEBOV and NiV.

### Genomic RNAs of segmented NSVs differ in their ability to trigger IFN induction

**Figure 3:**
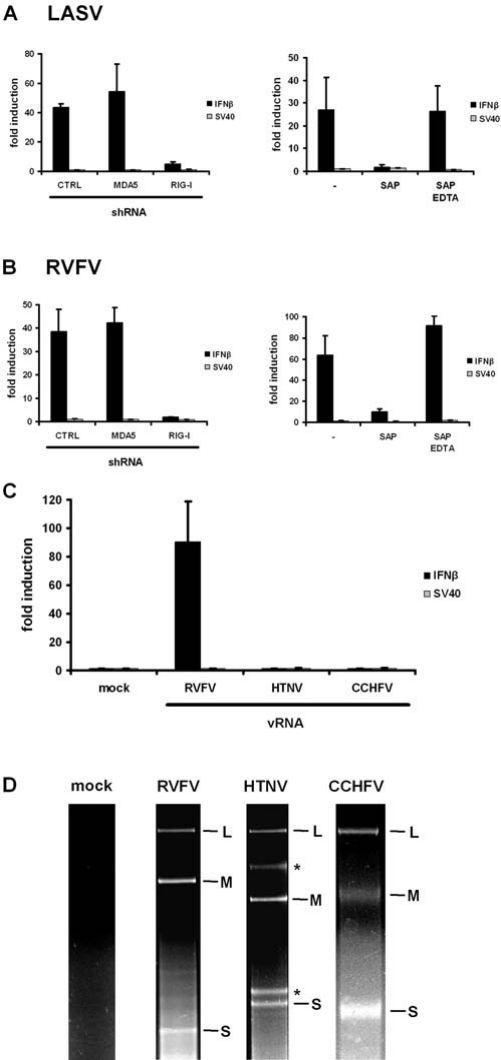
Genomic RNAs of segmented NSVs. RNAs isolated from virus particles of LASV (A) and RVFV (B) were tested for their ability to activate the IFN-β promoter in dependency of RIG-I or MDA5 (left panels) or 5′ triphosphate groups (right panels). (C) IFN-β promoter activation by genomic RNAs of the bunyaviruses RVFV, HTNV, and CCHFV. Mean values and standard deviations from 3 independent experiments are shown. (D) Purified virion RNAs (5 µg) of RVFV, HTNV, and CCHFV were separated on denaturing formaldehyde agarose gels. The genome segments (L, large; M, middle; S, small) are labeled on the right, and asterisks indicate 28S and 18S rRNA bands. Contamination of virus preparations with ribosomal RNAs are often observed [49] but have no consequences for IFN induction. As a control RNA isolated from ultracentrifuged supernatants of uninfected cells is shown (mock).

We extended our studies to NSV groups containing a segmented genome. As highly relevant representatives we investigated Lassa virus (LASV), a member of the *Arenaviridae* family, and several members of the *Bunyaviridae* family, namely Rift Valley fever virus (RVFV, genus phlebovirus), Hantaan virus (HTNV, genus hantavirus), and Crimean-Congo hemorrhagic fever virus (CCHFV, genus nairovirus). The RNAs of LASV and RVFV activated the IFN-β promoter in a RIG-I- and 5′ triphosphate-dependent manner as observed for the nonsegmented NSVs in the previous experiments (Fig. 3A and B). Surprisingly, however, RNAs isolated from HTNV and CCHFV particles did not trigger IFN induction (Fig. 3C). To rule out trivial explanations for the lack of stimulatory activity, we confirmed the identity and integrity of the bunyavirus genomic RNAs by RT-PCR analysis (see below) and denaturing formaldehyde agarose gel electrophoresis (Fig. 3D). Thus, in stark contrast to our findings with the other NSVs, the genomic RNAs of bunyaviruses belonging to the genera hantavirus and nairovirus are devoid of an IFN-inducing activity.

### Non-inducing viral RNAs contain a 5′ monophosphate

**Figure 4:**
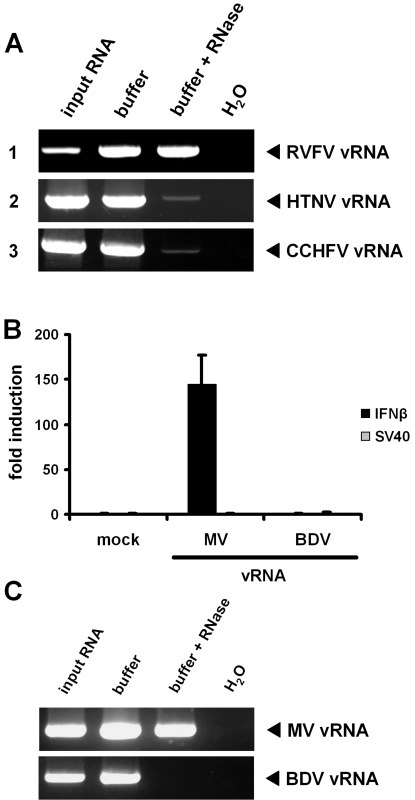
Non-inducing viral RNAs contain a 5′ monophosphate. (A) vRNAs of RVFV (panel 1), HTNV (panel 2) and CCHFV (panel 3) were incubated with a 5′monophosphate-specific 5′-3′ exonuclease. After 4 h of incubation, digestion efficacy was tested by RT-PCR analysis using primer pairs specific for the viral S segment. A comparison with untreated vRNAs is shown (lane input RNA). As additional controls, RNA was incubated without enzyme (lane buffer) or H_2_O was used for RT-PCR. The faint residual RT-PCR bands obtained after digestion of HTNV or CCHFV vRNAs are most likely caused by a minority of RNAs containing exonuclease-resistant 5′-OH ends. Such 5′-OH ends were previously observed for HTNV vRNA and thought to represent a preparation artifact [18]. (B) Activation of the IFN-β promoter by genomic RNAs isolated from MV and BDV particles. Mean values and standard deviations from 3 independent experiments are shown. (C) Treatment of MV and BDV genomic RNAs with a 5′ monophosphate-specific 5′-3′ exonuclease and subsequent RT-PCR analysis.

The absence of IFN induction by hantaviral and nairoviral RNA suggests a particular strategy to avoid RIG-I activation. As all initial products of RNA polymerases contain a triphosphorylated 5′ end [17], we hypothesised that those viruses which possess non-inducing genomic RNAs have removed the 5′ triphosphate group. In fact, for HTNV it was previously shown that the first nucleotide of the genome 5′ end is cleaved off by a viral endonuclease activity (“prime and realign”), resulting in a monophosphorylated 5′ end [18]. To investigate whether CCHFV may follow a similar strategy, we employed a 5′-3′ exonuclease which specifically digests ssRNA having a 5′ monophosphate [19]. As control, we incubated RVFV RNA which should be protected from enzymatic degradation due to its 5′ triphosphate group. Fig. 4A shows that RVFV RNA can still be detected after treatment with the 5′ monophosphate-specific RNase (panel 1), whereas HTNV RNA is degraded (panel 2). A similar RNase sensitivity was observed with RNA from CCHFV particles (panel 3). Thus, the inability of viral RNAs to activate RIG-I-dependent IFN induction correlates with the presence of a 5′ monophosphate.

An unusual mechanism of RNA replication was also proposed for one other member of the NSVs, Borna disease virus (BDV) [20], [21]. Interestingly, RNA isolated from BDV particles was unable to activate the IFN promoter, whereas RNA of measles virus (MV) was a good inducer, as expected (Fig. 4B). Moreover, BDV RNA, but not MV RNA, was undetectable after treatment with the 5′ monophosphate-specific RNase (Fig. 4C). Thus, BDV, the sole representative of the *Bornaviridae* family, avoids RIG-I activation in a manner similar to HTNV and CCHFV, namely by generating a 5′-terminal monophosphate during the course of genome replication.

### Non-inducing viral RNAs are not bound by RIG-I

**Figure 5:**
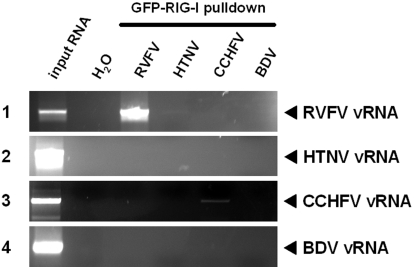
vRNA binding by RIG-I. GFP-RIG-I expressed by 293T cells was coupled to protein G Sepharose beads via a GFP-specific antiserum. Beads were incubated with vRNAs of either RVFV, HTNV, CCHFV, or BDV. After extensive washing, RNAs were extracted from the precipitates and cDNA synthesis was performed using random hexanucleotide oligomers. An aliquot of 10% of the input RNA was kept as RT-PCR control (first lane). All precipitated RNAs were subjected to RT-PCR specific for sequences of RVFV (panel 1), HTNV (panel 2), CCHFV (panel 3), and BDV (panel 4). H_2_O was used as negative control.

We wondered whether non-inducing vRNAs would differ from inducing vRNAs in the ability to bind RIG-I. To investigate this, we performed an RNA pulldown assay, using GFP-RIG-I coupled to Sepharose beads (see Materials and Methods). These GFP-RIG-I beads were incubated with vRNAs of either RVFV, HTNV, CCHFV, or BDV. After extensive washing, RNAs were isolated from the GFP-RIG-I beads and subjected to RT-PCR analysis. Fig. 5 (panel 1) shows that the 5′ triphosphate-containing vRNA of RVFV was precipitated by the GFP-RIG-I beads, as expected. SAP treatment abrogated RIG-I binding (data not shown), thus confirming the dependency of the interaction on the 5′-terminal triphosphate group. In contrast to RVFV, particle RNAs of HTNV, CCHFV and BDV were not bound by RIG-I (Fig. 5, panels 2 to 4). These data support the notion that processing of RNA 5′ ends enables viruses to escape RIG-I signaling and, hence, innate immune activation.

Taken together, our results demonstrate that the genomic RNAs from several highly pathogenic NSVs trigger RIG-I-dependent cytokine induction via their 5′-terminal triphosphate group, whereas MDA5 plays no apparent role. In addition, we have uncovered that members of the *Bunyaviridae* and *Bornaviridae* have evolved a unique strategy to escape this potent pathogen sensing mechanism by removing the 5′-terminal triphosphate group from their genome.

## Discussion

Viruses with a negative-stranded RNA genome are responsible for a wide range of diseases with significant medical and economical impact. They are divided into groups having a nonsegmented genome (*Paramyxoviridae*, *Filoviridae*, *Rhabdoviridae*, *Bornaviridae*) and those having a segmented genome (*Arenaviridae*, *Bunyaviridae*, *Orthomyxoviridae*). The genome replication strategy of NSVs involves primer-independent initiation of RNA synthesis by a single nucleotide. Due to this mechanism, all NSVs were believed to contain a triphosphate group at the 5′ ends of their genomic RNAs, with the notable exception of hantaviruses for which a “prime and realign” mechanism was described [18]. However, to our knowledge, IFN-inducing 5′ triphosphates have only been proven for the RNAs of rabies virus and vesicular stomatitis virus (family *Rhabdoviridae*) and FLUAV (family *Orthomyxoviridae*) [15], [16]. Here, we undertook a systematic study of all remaining NSV families and found that HTNV, CCHFV and BDV contain 5′ monophosphates whereas all other NSVs tested indeed had triphosphates. These analyses suggest that the 5′ triphosphate group may be a major determinant of the massive cytokine responses which are observed for infections with ZEBOV, NiV, LASV, and RVFV [9], [22]. By contrast, a recent study involving hantavirus-infected patients showed that type I IFN levels did not increase during the course of disease [23]. Moreover, IFN upregulation by CCHFV or BDV infection were not reported so far.

In the recent years, an increasing number of viral proteins have been described which are effectively downregulating the induction of IFN and other cytokines, the so-called IFN antagonists [4], [5]. Our results using genomic RNA from HTNV, CCHFV and BDV establish the generation of 5′-terminal monophosphates as a novel strategy to avoid the activation of the IFN system. Interestingly, the mechanisms of 5′ end processing differ between the viruses. HTNV employs prime-and realign to generate perfect complementary 5′ and 3′ genome ends able to form a panhandle structure, whereas BDV uses genome trimming resulting in a 3′end overhang [18], [20]. The strategy by which CCHFV processes its genome is currently unknown. However, viral RNA polymerases are only able to initiate RNA synthesis with a purine, but not with a pyrimidine [24], and CCHFV has 5′-terminal pyrimidine residues [25]. Moreover, for nairoviruses a previous study had suggested that polymerase slippage occurs during RNA synthesis [26]. These observations further support our claim that, for genome replication, nairoviruses utilize a similar prime and realign mechanism involving removal of the initiating 5′ nucleotide as hantaviruses do. The fact that the largely unrelated bunyaviruses and bornaviruses apply apparently different means to reach the same goal strongly suggests an independent evolution towards genomic 5′ monophosphates, driven by the selection pressure of RIG-I and the antiviral IFN system. Strikingly, both HTNV and BDV are known to establish persistent infection in their natural hosts [21], [27], and for CCHFV there is no evidence that it causes overt disease in carrier animals [7]. Possibly, the absence of a RIG-I ligand on the NSVs genome may facilitate the establishment of persistent or inapparent infection.

All NSV groups which were found to possess 5′ triphosphates, namely paramyxoviruses, rhabdoviruses, filoviruses, arenaviruses, orthobunya- and phleboviruses and orthomyxoviruses are known to encode IFN antagonists acting on the level of IFN induction [4], [28]–[34]. Moreover, the fact that ssRNA with a triphosphorylated 5′ end is an excellent trigger of RIG-I implies that an increase in the number of genome segments contained within infecting particles requires an increasingly strong and early anti-IFN function as a counterbalance. Indeed, orthobunyaviruses and phleboviruses, which have a tripartite genome, as well as the influenza A virus which has eight segments express potent IFN antagonists [4], [29], [35]. Interestingly, these segmented NSVs dedicate particular non-structural proteins (NSs for Bunyaviruses, NS1 for influenza virus) to silence the IFN system, whereas two-segmented and nonsegmented NSVs mostly employ structural proteins for this purpose [30]–[33]. This observation may implicate that for NSVs with few genome segments and hence few RIG-I ligands it is sufficient to endow structural proteins with an additional IFN-antagonistic function, whereas a higher number of genome segments requires either strong and specialized factors like NS1 and NSs, or the removal of the RIG-I ligands. Moreover, the fact that NSVs containing 5′ triphosphates elicit strong cytokine responses despite the presence of IFN-antagonistic proteins indicates that the balance between induction and viral inhibition is disturbed at some point during infection, possibly by a surplus of non-functional genomes such as produced by defective interfering particles [36] or by infection of cells which are resistant to the action of viral IFN antagonists.

In addition to the removal of the RIG-I ligand, hantaviruses modulate IFN induction by a weakly active NSs protein [37] and by an additional function of their glycoproteins [38], [39]. Likewise, the BDV P protein interferes with IFN induction [40], whereas for CCHFV no IFN antagonistic protein has been identified so far. The necessity to actively abrogate IFN induction despite the absence of the 5′triphosphate group may be due to the presence of other viral structures which trigger an innate immune response, e.g. viral nucleocapsids or traces of dsRNA which were shown to play a role in NSV cytokine activation [41]–[45]. Moreover, enveloped virus particles by themselves can activate IRF-3 in a TLR- and RIG-I-independent manner [46], [47], implicating that mammalian hosts can respond to several independent hallmarks of virus infection.

In summary, our study of the negative-stranded group of RNA viruses revealed that genome recognition by RIG-I is indeed a widespread phenomenon, but that hantaviruses, nairoviruses and bornaviruses avoid induction of antiviral responses by processing their RNA termini. These findings may help to better understand viral pathogenesis and particular cytokine profiles observed during infections.

## Materials and Methods

### Chemicals, cells and viruses

Puromycin (Sigma) was dissolved in H_2_O to 2 µg/µl and used at a concentration of 2 µg/ml cell culture medium. Polyethylen glycol (PEG) 8000 and poly(IC) were dissolved and used as indicated by the manufacturer (Sigma). Simian VeroE6 cells and human 293T cells were cultivated in Dulbecco's modified Eagle's medium (DMEM) supplemented with 10% FCS. Stocks of FLUAV strain PR/8/34 were prepared under BSL-2 conditions by culturing MDCKII cells in DMEM supplemented with 10% bovine serum albumin, containing 1 µg/ml trypsine. RVFV strain Clone 13, MV, and BDV were propagated in Vero cells under BSL-2 conditions. HTNV strain 76-118 was propagated in VeroE6 cells under BSL-3 conditions. NiV, ZEBOV strain Mayinga, LASV strain Josiah, and CCHFV strain IbAr10200 were propagated in VeroE6 cells under BSL-4 conditions.

### Plasmid constructs

The firefly luciferase reporter construct for monitoring IFN-β promoter activation (p125Luc) was kindly provided by Takashi Fujita, Kyoto University, Japan [48]. The control plasmid pRL-SV40 (Promega) contains the *Renilla* luciferase gene under control of the constitutively active SV40 promoter. Expression plasmids pHA-MDA5 encoding human MDA5 fused to an N-terminal HA tag and pGFP-RIG-I expressing human RIG-I fused to the C terminus of EGFP were described previously [16].

### Preparation of virus particles

Supernatants from infected cells were harvested at day 2 (CCHFV), day 3 (FLUAV, RVFV, NiV), day 4 (ZEBOV, MV), day 7 (LASV), day 10 (HTNV), or day 60 (BDV) post-infection. For preparation of virus particles, cell culture supernatants were first pre-cleared from cell debris by centrifuging at 1500 rpm for 10 min and 4°C. Then, virus particles were concentrated either by ultracentrifugation at 24,000 rpm for 2 h and 4°C (RVFV, MV, BDV), ultracentrifugation at 45,000 rpm for 3.5 h at 4°C (CCHFV, HTNV), or by PEG precipitation (FLUAV, NiV, ZEBOV, LASV). IFN-inducing properties are not affected by the method of virus concentration (data not shown). For PEG-mediated particle precipitation, 175 ml of pre-cleared cell culture supernatant were supplied with 12.25 g PEG 8000 and 4.02 g NaCl and stirred overnight at 4°C. Then, samples were transferred into 50 ml Falcons and centrifuged at 6,000 rpm for 45–60 min at 4°C. The pellet was dissolved in 1.5 ml of TriFast reagent (Peqlab). RNA isolation was performed as described by the manufacturer.

### Transient transfections and reporter gene assays

Subconfluent 293T cell monolayers grown in 12-well dishes were transfected with 0.25 µg p125-Luc and 0.05 µg pRL-SV40 in 100 µl OptiMEM (Gibco-BRL) containing 0.9 µl Fugene HD (Roche). After 6 h at 37°C, the liposome-DNA mixture was removed and cells were transfected either with 1 µg of viral RNA or with 5 µg of poly(IC) using 3 µl Metafectene (Biontex) per µg RNA prepared in 100 µl OptiMEM. At 18 h post transfection, cells were harvested and lysed in 100 µl of Passive Lysis Buffer (Promega). An aliquot of 20 µl lysate was used to measure luciferase activities using the dual luciferase assay (Promega).

### RT-PCR analysis

RNA was extracted using the TriFast reagent (Peqlab) and treated with DNase I. Reverse transcription was performed with 200 U of RevertAid H Minus M-MuLV (Fermentas) and 200 ng random hexanucleotides in 20 µl 1× M-MuLV RT reaction buffer supplied with 1 mM of each deoxynucleotide triphosphate (dNTP) and 40 U RNase Inhibitor (Fermentas). The resulting cDNA was amplified by 35 cycles of PCR, with each cycle consisting of 30 sec at 95°C, 30 sec at 55°C, and 90 sec at 68°C, followed by a final elongation step for 10 min at 68°C. Primer sequences are available upon request.

### shRNA knockdowns

Gene silencing by RNAi was achieved using shRNAs expressed by retroviruses (shRNAmir constructs, Open Biosystems). The construct which efficiently targets MDA5 had the catalog number RHS1764-9494563 (MDA5 #1) whereas RIG-I was targeted with RHS1764-9499511 (RIG-I #2). The control shRNA construct targets human Hsp 70 interacting protein and contained the sequence RHS1764-9500871. For establishing RNAi, 293T cells grown in 6-well dishes to 80% confluency were transfected with 1 µg each of pVpack-VSV G, pVpack-GP (Stratagene) and the shRNA construct, subcultured, incubated with Puromycin at day 3 post-transfection, and then incubated for another 2 to 3 days before usage.

### Enzymatic treatments of RNA

To remove 5′-terminal phosphates, purified RNAs were treated with 1 U of shrimp alkaline phosphatase (SAP) according to the protocol of the manufacturer (Roche). After incubation at 37°C for 10 min in the presence or absence of the inhibitor 1 mM EDTA, the enzyme was heat-inactivated at 65°C for 15 min.

Terminator™ (Epicentre Biotechnologies) is a 5′-3′ exonuclease able to digest ssRNA with a 5′ monophosphate. To determine the presence of a 5′ monophosphate on genomic RNAs isolated from virus particles, 500 ng of RNA were either mock treated or incubated with 1 U of Terminator exonuclease for 4 h at 30°C. The RNA was then purified using RNAeasy Mini Kit (QIAGEN), eluated in nuclease-free H_2_O and treated with 1 U DNase (Fermentas). To check for the presence of RNA, RT-PCR analysis was performed as indicated above.

### RNA pulldown analysis

Human 293T cells grown in 6-well dishes to 60% confluency were transfected with 1 µg pGFP-RIG-I per well. After 40 h of incubation, cells were washed with phosphate-buffered saline and lysed for 10 min at room temperature in 200 µl of RIPA buffer (50 mM Tris/HCl pH 7.5, 150 mM NaCl, 1% NP-40, complete protease inhibitor mix (Roche)). In parallel, 20 µl of a 50% slurry of protein G Sepharose in RIPA buffer were preadsorbed with 5 µl polyclonal rabbit anti-GFP antiserum (Invitrogen) and incubated for 2 h at 4°C. Then, the Sepharose beads were washed three times with RIPA buffer and incubated for 2 h at 4°C with the lysate equivalent of half a 6-well plate of transfected 293T cells. The immunoprecipitates were washed three times with RIPA buffer and then incubated with 1 µg of viral RNA for a further 2 h at 4°C. After three washing steps using RIPA buffer, the volume of the Sepharose beads was adjusted with H_2_O to 100 µl and RNA was extracted using the RNeasy kit (Qiagen). RNA pulldown was analysed by RT-PCR using random hexamers for RT and virus-specific primers for PCR.

## References

[1] Bowie AG, Fitzgerald KA (2007). RIG-I: tri-ing to discriminate between self and non-self RNA.. Trends Immunol.

[2] Pichlmair A, Reis ESC (2007). Innate Recognition of Viruses.. Immunity.

[3] Iwasaki A, Medzhitov R (2004). Toll-like receptor control of the adaptive immune responses.. Nat Immunol.

[4] Garcia-Sastre A, Biron CA (2006). Type 1 interferons and the virus-host relationship: a lesson in detente.. Science.

[5] Haller O, Kochs G, Weber F (2006). The interferon response circuit: induction and suppression by pathogenic viruses.. Virology.

[6] Eaton BT, Broder CC, Middleton D, Wang LF (2006). Hendra and Nipah viruses: different and dangerous.. Nat Rev Microbiol.

[7] Ergonul O (2006). Crimean-Congo haemorrhagic fever.. Lancet Infect Dis.

[8] Feldmann H, Jones S, Klenk HD, Schnittler HJ (2003). Ebola virus: from discovery to vaccine.. Nat Rev Immunol.

[9] Geisbert TW, Jahrling PB (2004). Exotic emerging viral diseases: progress and challenges.. Nat Med.

[10] Hiscott J (2007). Triggering the innate antiviral response through IRF-3 activation.. J Biol Chem.

[11] Andrejeva J, Childs KS, Young DF, Carlos TS, Stock N (2004). The V proteins of paramyxoviruses bind the IFN-inducible RNA helicase, mda-5, and inhibit its activation of the IFN-beta promoter.. Proc Natl Acad Sci U S A.

[12] Kato H, Takeuchi O, Sato S, Yoneyama M, Yamamoto M (2006). Differential roles of MDA5 and RIG-I helicases in the recognition of RNA viruses.. Nature.

[13] Yoneyama M, Fujita T (2007). Function of RIG-I-like receptors in antiviral innate immunity.. J Biol Chem.

[14] Weber F, Wagner V, Rasmussen SB, Hartmann R, Paludan SR (2006). Double-stranded RNA is produced by positive-strand RNA viruses and DNA viruses but not in detectable amounts by negative-strand RNA viruses.. J Virol.

[15] Hornung V, Ellegast J, Kim S, Brzozka K, Jung A (2006). 5′-Triphosphate RNA Is the Ligand for RIG-I.. Science.

[16] Pichlmair A, Schulz O, Tan CP, Naslund TI, Liljestrom P (2006). RIG-I-Mediated Antiviral Responses to Single-Stranded RNA Bearing 5′ Phosphates.. Science.

[17] Schlee M, Barchet W, Hornung V, Hartmann G (2007). Beyond double-stranded RNA-type I IFN induction by 3pRNA and other viral nucleic acids.. Curr Top Microbiol Immunol.

[18] Garcin D, Lezzi M, Dobbs M, Elliott RM, Schmaljohn C (1995). The 5′ ends of Hantaan virus (Bunyaviridae) RNAs suggest a prime-and-realign mechanism for the initiation of RNA synthesis.. J Virol.

[19] Pak J, Fire A (2007). Distinct populations of primary and secondary effectors during RNAi in C. elegans.. Science.

[20] Schneider U, Schwemmle M, Staeheli P (2005). Genome trimming: a unique strategy for replication control employed by Borna disease virus.. Proc Natl Acad Sci U S A.

[21] Schneider U, Martin A, Schwemmle M, Staeheli P (2007). Genome trimming by Borna disease viruses: Viral replication control or escape from cellular surveillance?. Cell Mol Life Sci.

[22] Kash JC, Muhlberger E, Carter V, Grosch M, Perwitasari O (2006). Global suppression of the host antiviral response by Ebola- and Marburgviruses: increased antagonism of the type I interferon response is associated with enhanced virulence.. J Virol.

[23] Stoltz M, Ahlm C, Lundkvist A, Klingstrom J (2007). Lambda Interferon (IFN-{lambda}) in Serum Is Decreased in Hantavirus-Infected Patients, and In Vitro-Established Infection Is Insensitive to Treatment with All IFNs and Inhibits IFN-{gamma}-Induced Nitric Oxide Production.. J Virol.

[24] Banerjee AK (1980). 5′-terminal cap structure in eucaryotic messenger ribonucleic acids.. Microbiol Rev.

[25] Elliott RM (1996). The Bunyaviridae.

[26] Jin H, Elliott RM (1993). Non-viral sequences at the 5′ ends of Dugbe nairovirus S mRNAs.. J Gen Virol.

[27] Meyer BJ, Schmaljohn CS (2000). Persistent hantavirus infections: characteristics and mechanisms.. Trends Microbiol.

[28] Billecocq A, Spiegel M, Vialat P, Kohl A, Weber F (2004). NSs protein of Rift Valley fever virus blocks interferon production by inhibiting host gene transcription.. J Virol.

[29] Blakqori G, Delhaye S, Habjan M, Blair CD, Sanchez-Vargas I (2007). La Crosse bunyavirus nonstructural protein NSs serves to suppress the type I interferon system of mammalian hosts.. J Virol.

[30] Brzozka K, Finke S, Conzelmann KK (2005). Identification of the rabies virus alpha/beta interferon antagonist: phosphoprotein P interferes with phosphorylation of interferon regulatory factor 3.. J Virol.

[31] Cardenas WB, Loo YM, Gale M, Hartman AL, Kimberlin CR (2006). Ebola virus VP35 protein binds double-stranded RNA and inhibits alpha/beta interferon production induced by RIG-I signaling.. J Virol.

[32] Martinez-Sobrido L, Zuniga EI, Rosario D, Garcia-Sastre A, de la Torre JC (2006). Inhibition of the type I interferon response by the nucleoprotein of the prototypic arenavirus lymphocytic choriomeningitis virus.. J Virol.

[33] Shaw ML, Cardenas WB, Zamarin D, Palese P, Basler CF (2005). Nuclear localization of the Nipah virus W protein allows for inhibition of both virus- and toll-like receptor 3-triggered signaling pathways.. J Virol.

[34] Weber F, Bridgen A, Fazakerley JK, Streitenfeld H, Randall RE (2002). Bunyamwera bunyavirus nonstructural protein NSs counteracts the induction of alpha/beta interferon.. J Virol.

[35] Le May N, Dubaele S, De Santis LP, Billecocq A, Bouloy M (2004). TFIIH transcription factor, a target for the Rift Valley hemorrhagic fever virus.. Cell.

[36] Strahle L, Garcin D, Kolakofsky D (2006). Sendai virus defective-interfering genomes and the activation of interferon-beta.. Virology.

[37] Jääskeläinen KM, Kaukinen P, Minskaya ES, Plyusnina A, Vapalahti O (2007). Tula and Puumala Hantavirus NSs ORFs are Functional and the Products Inhibit Activation of the Interferon-Beta Promoter.. J Med Virol.

[38] Alff PJ, Gavrilovskaya IN, Gorbunova E, Endriss K, Chong Y (2006). The pathogenic NY-1 hantavirus G1 cytoplasmic tail inhibits RIG-I- and TBK-1-directed interferon responses.. J Virol.

[39] Spiropoulou CF, Albarino CG, Ksiazek TG, Rollin PE (2007). Andes and Prospect Hill Hantaviruses Differ in Early Induction of Interferon although Both Can Downregulate Interferon Signaling.. J Virol.

[40] Unterstab G, Ludwig S, Anton A, Planz O, Dauber B (2005). Viral targeting of the interferon-beta inducing Traf family member-associated NF-kB activator-(TANK) binding kinase-1.. PNAS.

[41] Le Goffic R, Balloy V, Lagranderie M, Alexopoulou L, Escriou N (2006). Detrimental contribution of the Toll-like receptor (TLR)3 to influenza A virus-induced acute pneumonia.. PLoS Pathog.

[42] Le Goffic R, Pothlichet J, Vitour D, Fujita T, Meurs E (2007). Cutting Edge: Influenza A Virus Activates TLR3-Dependent Inflammatory and RIG-I-Dependent Antiviral Responses in Human Lung Epithelial Cells.. J Immunol.

[43] Liu P, Jamaluddin M, Li K, Garofalo RP, Casola A (2007). Retinoic acid-inducible gene I mediates early antiviral response and Toll-like receptor 3 expression in respiratory syncytial virus-infected airway epithelial cells.. J Virol.

[44] tenOever BR, Servant MJ, Grandvaux N, Lin R, Hiscott J (2002). Recognition of the measles virus nucleocapsid as a mechanism of IRF-3 activation.. J Virol.

[45] tenOever BR, Sharma S, Zou W, Sun Q, Grandvaux N (2004). Activation of TBK1 and IKK epsilon kinases by vesicular stomatitis virus infection and the role of viral ribonucleoprotein in the development of interferon antiviral immunity.. J Virol.

[46] Paladino P, Cummings DT, Noyce RS, Mossman KL (2006). The IFN-independent response to virus particle entry provides a first line of antiviral defense that is independent of TLRs and retinoic acid-inducible gene I.. J Immunol.

[47] Prescott JB, Hall PR, Bondu-Hawkins VS, Ye C, Hjelle B (2007). Early innate immune responses to sin nombre hantavirus occur independently of IFN regulatory factor 3, characterized pattern recognition receptors, and viral entry.. J Immunol.

[48] Yoneyama M, Suhara W, Fukuhara Y, Fukuda M, Nishida E (1998). Direct triggering of the type I interferon system by virus infection: activation of a transcription factor complex containing IRF-3 and CBP/p300.. Embo J.

[49] Staunton D, Nuttall PA, Bishop DH (1989). Sequence analyses of Thogoto viral RNA segment 3: evidence for a distant relationship between an arbovirus and members of the Orthomyxoviridae.. J Gen Virol.

